# Flow Cytometry as a Tool for Quality Control of Fluorescent Conjugates Used in Immunoassays

**DOI:** 10.1371/journal.pone.0167669

**Published:** 2016-12-09

**Authors:** Marta de Almeida Santiago, Bruna de Paula Fonseca e Fonseca, Christiane de Fátima da Silva Marques, Edimilson Domingos da Silva, Alvaro Luiz Bertho, Ana Cristina Martins de Almeida Nogueira

**Affiliations:** 1 Laboratory of Diagnostic Technology, Immunobiological Technology Institute, FIOCRUZ, Rio de Janeiro, Rio de Janeiro, Brazil; 2 Laboratory of Immunoparasitology, Oswaldo Cruz Institute, FIOCRUZ, Rio de Janeiro, Rio de Janeiro, Brazil; 3 Flow Cytometry Core Facility, Oswaldo Cruz Institute, FIOCRUZ, Rio de Janeiro, Rio de Janeiro, Brazil; 4 Laboratory of Clinical Immunology, Oswaldo Cruz Institute, FIOCRUZ, Rio de Janeiro, Rio de Janeiro, Brazil; Universidade de Sao Paulo Instituto de Quimica, BRAZIL

## Abstract

The use of antibodies in immunodiagnostic kits generally implies the conjugation of these proteins with other molecules such as chromophores or fluorochromes. The development of more sensitive quality control procedures than spectrophotometry is essential to assure the use of better fluorescent conjugates since the fluorescent conjugates are critical reagents for a variety of immunodiagnostic kits. In this article, we demonstrate a new flow cytometric protocol to evaluate conjugates by molecules of equivalent soluble fluorochromes (MESF) and by traditional flow cytometric analysis. We have coupled microspheres with anti-IgG-PE and anti-HBSAg-PE conjugates from distinct manufactures and/or different lots and evaluated by flow cytometry. Their fluorescence intensities were followed for a period of 18 months. Our results showed that there was a great difference in the fluorescence intensities between the conjugates studied. The differences were observed between manufactures and lots from both anti-IgG-PE and anti-HBSAg-PE conjugates. Coefficients of variation (CVs) showed that this parameter can be used to determine better coupling conditions, such as homogenous coupling. The MESF analysis, as well as geometric mean evaluation by traditional flow cytometry, showed a decrease in the values for all conjugates during the study and were indispensable tools to validate the results of stability tests. Our data demonstrated the feasibility of the flow cytometric method as a standard quality control of immunoassay kits.

## Introduction

Monoclonal antibodies are glycoproteins containing uniform variable regions that confer a high specificity for a single epitope [1], favoring their use not only in scientific research, but also in immunodiagnostic and therapy. In basic research, they are primarily used for staining both surface and intracellular proteins, like membrane receptors and cytokines [2]. In therapy, there are numerous monoclonal antibodies licensed for the treatment of various diseases, like cancer, allergy and autoimmune diseases [3–5]. The use of antibodies in immunodiagnostic kits generally implies the conjugation of these proteins with other molecules, such as chromophores or fluorochromes (i.e. phycoerythrin—PE or fluorescein isothiocyanate—FITC), that make the reaction detectable. Those kits are applied to detect several types of molecules, such as drugs, hormones, infectious disease biomarkers and other types of antigens on antibody-based multiplex, enzyme-linked immunosorbent (ELISA) or flow cytometry assays [6]. Hence, the credibility of the results obtained in this type of assays strongly depends on the conjugates performance.

The quality control of fluorescent conjugates is usually performed by spectrophotometry, where the ratio between fluorochrome and protein (F/P ratio) is measured. This ratio is determined by reading the optical densities (OD) of the antibody and fluorochromes in the spectrophotometer. After the conjugation process, the conjugates have to achieve their ideal F/P ratio determined in the conjugation protocol, which varies depending on the fluorochrome that is used. However, according to Vogt *et al*. [7], the F/ P ratio does not necessarily express the fluorescence emission. Since the latest depends on the sort of energy excitation, which in a spectrometer is not present, one might have a good F/P ratio for a molecule, but not necessarily a satisfactory emission of the same molecule when it is tested in a flow cytometer. Therefore, these conjugates can compromise the results from flow cytometry as well as from other technologies.

Regardless the technology applied for the evaluation of these conjugates, the need for better quality control tools raises as their application in different processes increases. In fact, several studies have pointed out the need for the development of more sensitive techniques and the importance of quality programs to obtain satisfactory results in clinical laboratories [8–10]. According to Ellington *et al*. [11] there are few reagents and procedures for quality control testing in antibody-based multiplex technology and there is an imperative need to develop appropriate analytical validation and quality control procedures so that this technology can reach the *in vitro* diagnostic market with a safety assurance.

As mentioned above, flow cytometry is one of the technologies that mainly rely on conjugates. This technology has been used as an important tool in basic research, clinical diagnosis of hematopoietic syndromes, potency assays, sanitary, environmental and food microbiology, alternative tests for animal use and others [2, 12–15]. In quality control, numerous applications have been proposed with flow cytometry in the monitoring of products as well as processes from the food industry [16], immunotherapeutic products [17, 18] and protocols and assays in clinical laboratories [8, 10]. In the traditional flow cytometric analysis, fluorescence intensity is evaluated based on data expressed in geometric means, coefficient of variation (CV) and percentage. These three parameters can provide different information about the fluorescence of each conjugate: the mean confers the intensity of the sign detected by the photomultiplier and for this reason can express the brightness of the fluorochrome; the CV can be used to express the homogeneity of the staining as narrow peaks presents lower CVs; and the percentage represents the number of positive cells or particles for the target molecule. Besides the traditional analysis, quantitative fluorescence cytometry (QFCM) has been proposed as an alternative to these measurements [19, 20]. The QFCM uses microspheres coupled with different amounts of fluorochrome to measure the fluorescent intensity of unknown labeled particles [20]. Such measurements are made by evaluating the values of molecules of equivalent soluble fluorochromes (MESF), expressed as the number of fluorochrome molecules in solution required to produce the same intensity of fluorescence measured in the labeled particle. MESF studies are based on the equivalence between the number of fluorochromes in two solutions, where one can be a suspension of labeled microspheres and the other an unknown sample [21]. The applications of QFCM include calibration and linearity verification of instruments [22] as well as research and diagnostic studies [23–25]. Despite the different techniques proposed for this quantitative evaluation, there is a consensus on the requirement of efficient standardization of the methodology with respect to instruments and reagents (antibodies and microspheres), since at least for reagents the limit of detection will be necessarily fixed at the highest possible population of the MESF kits applied [26, 27].

In this article we present a novel flow cytometric method to evaluate conjugates using microspheres coupled with different PE-labeled antibodies by the traditional flow cytometric analysis and by QFCM. We used the geometric mean of PE histograms to evaluate the conjugates’ brightness and stability and the CV of positive PE peaks to evaluate homogeneity of the coupling process of the conjugates to the beads. Besides that, we also assessed the feasibility of these analyses on the evaluation of conjugate stability. For this purpose, the study was designed using PE conjugates of different lots and manufactures, the chosen reagent in antibody-based multiplex technology assays, with the aim to establish method conditions as well as to assure the feasibility of flow cytometry in the detection of possible signal variations.

## Materials and Methods

### Conjugates

Goat anti-human IgG (ɤ-chain specific)-R-phycoerythrin (anti-IgG-PE) conjugates were purchased from two different manufacturers (Sigma, catalog number P9170; MOSS, catalog number GTIG-001). Four different lots of Sigma conjugates (A1- lot number 031M6086; A2- lot number 060M6024; A3- lot number 079K6032; A4- lot number SLBC5035V) and two different lots from MOSS (B1- lot number 02040091 and B2- lot number 082201111) were used. The anti-HBSAg conjugates (anti-HBSAg-PE) were obtained from two different manufacturers named C (Fitzgerald, catalog number 10-H05G, lot number 4176) and D (CalBio Reagents, catalog number P171, lot number PA427) conjugated to PE fluorochrome (Invitrogen—Molecular Probes, catalog number P801, lot numbers 699300 and 989639, respectively). These PE antibodies were chosen since they were used as reporter conjugates in multiplex assays in Luminex 100^™^ system (Luminex Corporation, Texas, USA). The conjugate best concentrations (0.5, 1, 2.5 and 5 μg/mL) or dilutions (1:3000, 1:1000 and 1:300) were determined according to the immunoassay protocol described elsewhere [28]. Stock solution concentrations of C and D conjugates, as obtained by spectrophotometry, were 1 mg/mL and 2.7 mg/mL respectively.

### Beads

The beads were 6-micron polystyrene paramagnetic carboxylated (MagPlex^™^ -C) microspheres obtained from Luminex Corp. (Texas, USA). They have a combination of two internal dyes, which is specific for each bead set. This combination results in a unique bead code number and fluorescent intensity signature. Different bead codes (012- lot number B18738; 027- lot number B23910; 035- lot number B21752; 039- lot number B20966; 065- lot numbers B15928 and B22601; 070- lot number B18006) were used and the possible interference of the internal dyes fluorescence on the detection of conjugate fluorescence was tested.

### Bead coupling to conjugates

The coupling of the conjugates to paramagnetic carboxylated beads was performed according to the manufacturer’s instructions. Briefly, the beads were vortexed and sonicated (2 cycles) in order to avoid aggregates and to ensure homogenous bead distribution in the solution. After that, 80 μl of the bead suspension (10^6^ beads) were washed twice with double-distilled water (ddH_2_O) and suspended in 80 μL of activation buffer containing 100 mM sodium phosphate (pH 6.2), 10μL of N-hydroxysulfosuccinimide (sulfo-NHS; Thermo, catalog number 24510) and 10 μL of 1-ethyl-3-(3-dimethylaminopropyl)-carbodiimide hydrochloride (EDC; Thermo, catalog number 22980), both diluted to a concentration of 50 mg/mL in ddH_2_O. They were added to stabilize the reaction and activate the beads. After mixing, beads were incubated for 20 minutes protected from light at room temperature. The activated beads were washed with coupling buffer [phosphate-buffered saline (PBS)] and 100 μL of conjugate solutions of each concentration and dilution tested were added. After 2 hours of incubation on a shaker (600 rpm) at room temperature, the beads were washed with wash/storage buffer [PBS, 1% bovine serum albumin (Amresco, catalog number 0332-100G), 0.02% Tween 20 (Merck, catalog number 8.22184.0500), 0.05% sodium azide (Merck, catalog number 1.06688.0100)] and suspended in 1 mL of the same buffer. The beads were stored protected from light at 2°C to 8°C until use.

In order to differentiate the loss of fluorescence emission of the fluorophore from protein degradation and uncoupling, beads coupled with conjugates were submitted to a secondary staining with rabbit anti-goat IgG-FITC (Sigma, catalog number F2016, lot number 053M4824V). First, it was evaluated the best concentration of anti-IgG-FITC to stain anti-IgG-PE coupled to the beads. For that purpose, microspheres coupled with A1 and A4 conjugates were stained with serial dilutions of anti-IgG-FITC (1:100, 1:200, 1:400, 1:800 and 1:1600). For dilution test and staining experiments of anti-IgG-FITC the following protocol was used: beads were vortexed and sonicated (2 cycles), washed with ddH_2_O (1:9) and suspended in 80 μL of wash buffer. The buffer was aspirated and 100 μl of anti-IgG-FITC were added to the beads, according to the experimental design. The beads were incubated for 15 minutes on a shaker (600 rpm) at 37°C and protected from light. After one wash cycle with storage buffer, supernatant was discarded and samples were suspended in 200 μl of sheath fluid (Isogen, Biotek) for analysis in the flow cytometer. In order to discriminate between PE fluorescence fading and uncoupling of the anti-IgG-PE conjugates from the beads, aliquots of those beads were maintained at room temperature and exposed to light for two weeks. As controls, we used beads not coupled with conjugates or coupled but incubated in the absence of anti-IgG-FITC.

### Flow cytometry

Flow cytometric analyses were performed with a FACSCalibur [Becton Dickinson (BD)] equipped with an argon laser (488nm) and a diode red laser (635nm). For data acquisition, the PE emission from conjugates was measured in the FL2 channel (band pass filter 585/42). Bead code identification was done in FL4 channel (band pass 661/16). FITC emission of anti-IgG-FITC staining was measured in the FL1 channel (band pass 530/30). The acquisition protocol used CellQuest software (BD) while data analyses were performed using FlowJo (Tree Star) and Kaluza 1.3 (Beckman Coulter) softwares. All evaluations were based on dot plots from forward scatter (FSC) and side scatter (SSC) and from histograms generated by PE or FITC emission. For each sample, the conjugate coupled beads were vortexed and sonicated and 16 μL of each solution were added to 200 μL of Isogen. A minimum of 2,000 events in the gate corresponding to the bead population on the FSC x SSC dot plots were analyzed.

Besides original flow cytometric analysis, in which parameters as geometric mean and coefficient of variation were evaluated, quantitative fluorescence cytometry (QFCM) was also performed using Quantum^™^ R-PE MESF (Bangs Laboratories, catalog number 827), according to the manufacturer’s instructions. The Quantum MESF kit contains five populations of fluorescent standard beads: four populations having different levels of PE fluorescence intensity and a Certified Blank^™^. Briefly, the conjugate coupled beads were vortexed, sonicated and 16 μL of each solution were added to 200 μL of Isogen. Immediately after the acquisition of the sample tubes, the MESF tube was acquired. This tube contained 500 μL of Isogen and one drop (approximately 40 μL) of each calibrated fluorescent standard beads of the Quantum MESF kit. All flow cytometer settings were maintained throughout the acquisitions. The geometric mean of the MESF tube and of the sample tubes were plotted in the Bangs Laboratories’ quantitative software, QuickCal, and the MESF values were analyzed.

Instrument quality control was performed, as usual, using BD CaliBRITE 3 Beads (BD, catalog number 340486), CaliBRITE APC (BD, catalog number 340487) and FACSComp software (BD) once a week during the whole experiment period. Instrument set up was established at the first experiment by positioning bead 1 of MESF kit between 10^0^ and 10^1^ and settings were maintained for all experiments. All five MESF populations were used in the analysis of conjugate and instrument performance.

### Statistical analysis

Statistical evaluations were performed using Prism software (GraphPad Inc.). Comparisons between groups and analysis of group variation were performed using T test and ANOVA, respectively. Significance was considered when p≤ 0.05.

## Results

### Influence of different codes of microspheres in the detection of PE fluorescence intensities

**Fig 1:**
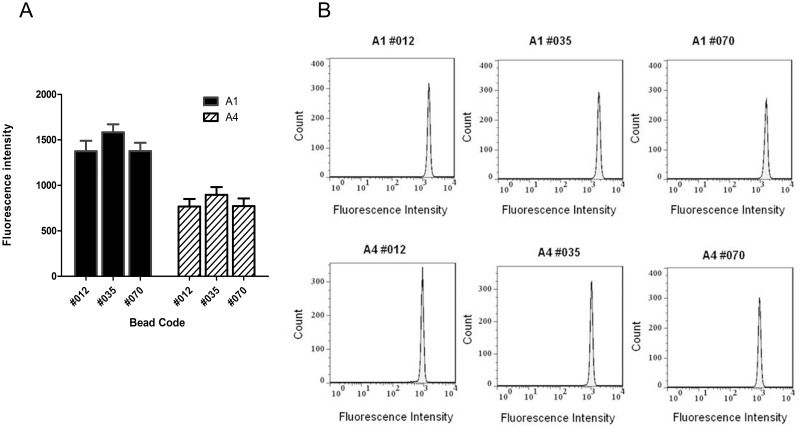
Evaluation of different codes of microspheres (#012, #035 and #070). A: Microspheres (#012, #035 and #070) coupled with 5 μg/mL of A1 (black bars) and A4 (dashed bars) conjugates. Fluorescence intensity represented by the means of three geometric means ± standard deviation. B: Representative histograms of one experiment. PE-fluorescence intensity (X axis) vs. count (Y axis). One way ANOVA was performed for group comparison and significant differences were observed among all groups (p≤0.05).

In order to assess whether different codes of microspheres might interfere with the evaluation of PE intensity, three different microsphere codes (#012, #035 and #070) were coupled with 5 μg/mL of A1 and A4 conjugates and evaluated by flow cytometry. The results showed that geometric means of fluorescence intensities were similar within the group A1 and A4 irrespective of the bead code used. Furthermore, when comparing the overall behavior of the conjugates, we observed that the fluorescence intensity mean of A1 was approximately 60% higher than the same parameter of A4 (Fig 1A). There was no difference in the peak profile of histograms for any of the bead codes studied (Fig 1B). A shift to the left was observed in the histogram of the A4 conjugates when compared to the A1 histograms. This was due to the lower brightness of A4 conjugate (Fig 1B).

### Evaluation of the optimal concentrations of conjugates for flow cytometric analysis

**Fig 2:**
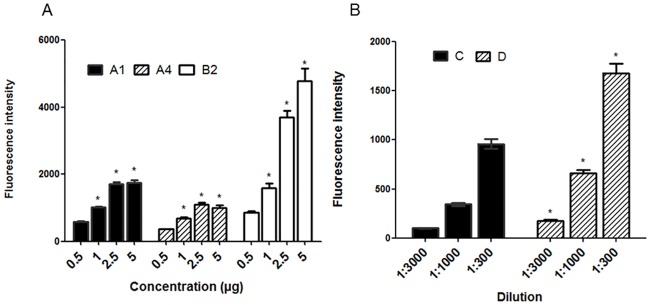
Evaluation of the best conjugate concentration and dilution. A: Anti-IgG-PE concentration test (0.5, 1, 2.5 and 5 μg/mL). * = statistically different value when compared to the lowest concentration (p≤0.05). A1- black bars, A4- dashed bars and B2- white bars. B: Anti-HBSAg-PE dilution test (1:3000, 1:1000 and 1:300). C- black bars and D- dashed bars. One way ANOVA was performed for group comparison * = statistically different value among concentrations (p≤0.05). Data are expressed as mean of three geometric means ± standard deviation.

The next step was to evaluate the best concentrations of anti-IgG-PE and the dilutions of anti-HBSAg-PE to be used for coupling. For this purpose, microspheres were coupled with different concentrations and dilutions of the conjugates and were then evaluated by flow cytometry. Anti-IgG-PE conjugates (A1, A4 and B2) were tested at concentrations of 0.5, 1, 2.5 and 5 μg/mL, while the anti-HBSAg-PE conjugates (C and D) were tested at dilutions 1:3000, 1:1000 and 1:300 from the stock solution. The fluorescence intensities of the anti-IgG-PE conjugates raised significantly with the increase on the concentrations (Fig 2A). On the other hand, the lowest dilution of the anti-HBSAg-PE conjugates resulted in the highest fluorescence intensity mean (Fig 2B). These observations led us to define optimal working concentration and dilution of 5 μg/mL and 1:300, respectively.

### Coefficient of variation as a tool to determine optimal conjugate concentration

**Fig 3:**
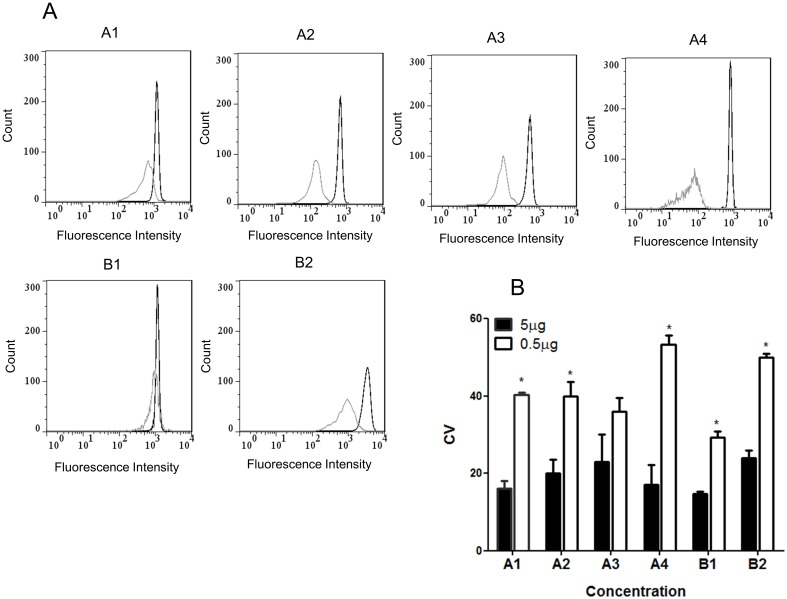
Staining profile of anti-IgG-PE conjugates. A: Representative histograms of PE-fluorescence intensity (X axis) vs. count (Y axis). 0.5 μg/mL = white peak and 5 μg/mL = black peak. B: Coefficient of variation of 0.5 μg/mL (white bars) and 5 μg/mL (black bars). Data expressed as the means of CVs from of three tests ± standard deviation. T test was performed for group comparison. An asterisk indicates a significantly different value assumed at p≤0.05.

In order to evaluate whether the concentration of anti-IgG-PE conjugates could influence on the homogeneity of profiles observed, we compared the highest and the lowest concentration of the coupling procedure and calculated the coefficients of variation (CVs) of each one. These results are shown in Fig 3A, where the highest concentration of 5 μg/mL (black peak) presented the narrowest peaks, while the lowest concentration of 0.5 μg/mL of the same conjugates had wider peaks (white peak, Fig 3A). Thus, the lowest CVs observed for the highest concentration of the conjugates demonstrate homogeneous couplings, indicating that 5 μg/mL was the optimal concentration for this matter (Fig 3B). Regarding the anti-HBSAg-PE conjugates, similar staining profiles and CVs were observed for all dilutions evaluated (data not shown).

### QFCM analysis of microspheres coupled with PE conjugates

**Fig 4:**
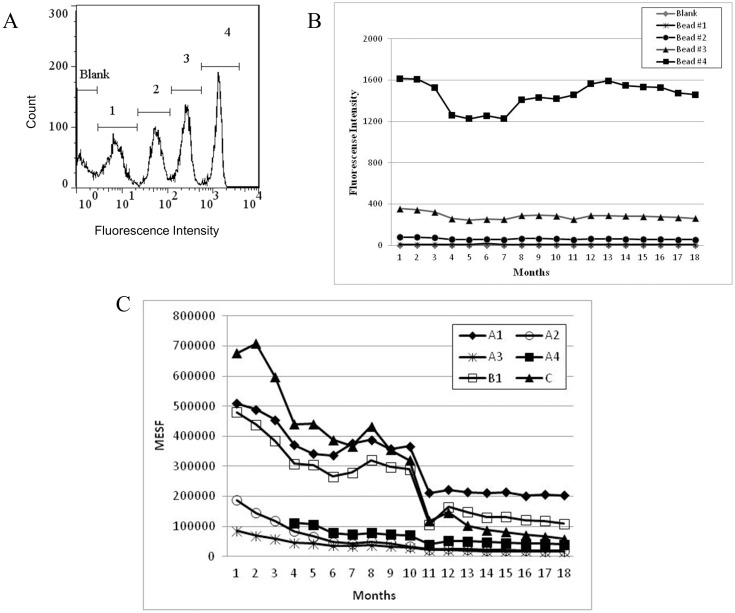
QFCM evaluation of anti-IgG-PE and anti-HBSAg-PE conjugates. A: Histogram of PE-fluorescence intensity (X axis) vs. count (Y axis) of MESF kit (populations blank, bead #1, bead #2, bead #3 and bead #4). B: Geometric means of MESF kit beads along the months. C: MESF values of A1, A2, A3, A4, B1 and C conjugates along the months. A4 conjugate was coupled later than the other conjugates and its intensities evaluation started at month 4.

After determining the optimal concentration and dilution of the conjugates, we initiated the studies of QFCM using the Quantum^™^ R-PE MESF kit. Beads with different fluorescence intensities were measured and optimal instrument settings were established (Fig 4A). Subsequently, the geometric means of beads coupled with conjugates were compared, at each point of analysis, with the geometric means of populations obtained in the MESF kit. During 18 months, beads #1 and #2 had fluorescence intensity means (geometric means) above 62, while beads #3 and #4 had approximately 278 and 1443 fluorescence intensity means, respectively (Fig 4B). Fig 4C shows the MESF values for A1, A2, A3, A4, B1 and C conjugates during the 18-month evaluation period, in which a decrease in the values of the conjugates relative to the MESF for all conjugates was observed. MESF beads brightness showed a decrease between the 4^th^ and 7^th^ month of the observation period, returning to initial conditions afterwards. Moreover, two conjugate fluorescence intensities, B2 and D, exceeded the maximum limit of the MESF kit and, therefore, could not be evaluated by QFCM.

In order to verify whether the variations observed for the conjugates values relative to MESF were due to uncoupling or antibody degradation, beads coupled with PE-conjugates submitted to fluorochrome degradation by light/temperature exposure were labeled with anti-IgG-FITC. Emission of both fluorochromes was, then, analyzed. As expected, samples submitted to room temperature and light showed an intense decrease of PE and no significant alteration on FITC emission when compared to samples protected from light and temperature (Table 1). Hence, an eventual uncoupling or antibody degradation, under these coupling conditions, did not result in a significant emission decrease.

**Table 1:** FITC and PE detection after anti-IgG-FITC staining.

	FITC Detection (FL1)		PE Detection (FL2)	
SAMPLES	2 a 8°C	RT^a^	2 a 8°C	RT
**#070**	2	X^b^	4	X
**A1**	80	71	731	56
**A2**	185	166	580	45
**A3**	131	118	366	35
**A4**	129	101	399	37

^a^ RT–room temperature.

^b^ X- not performed.

### Evaluation of performance and stability studies of PE conjugates

**Fig 5:**
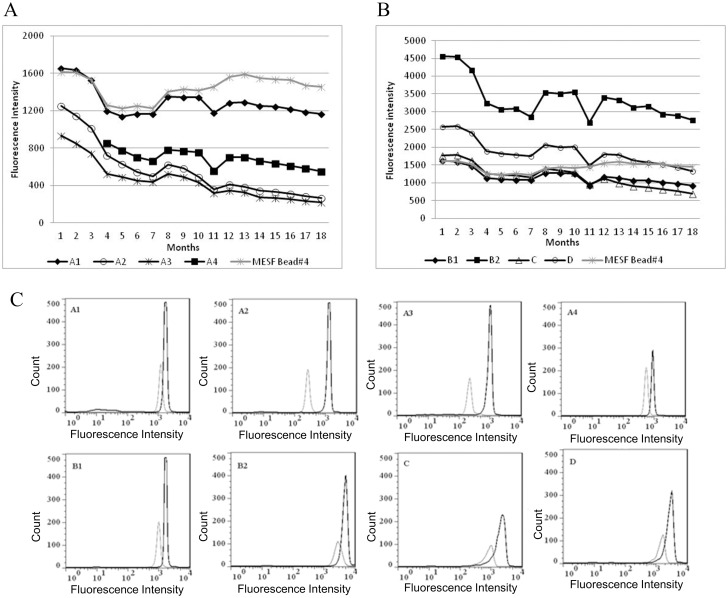
Conjugate stability study during 18 months. A: A1, A2, A3, A4 and MESF bead #4 fluorescence intensities during the months of analysis. B: B1, B2, C, D and MESF bead #4 fluorescence intensities during the months of analysis. C: Conjugates histograms of PE-fluorescence intensity (X axis) vs. count (Y axis) on the first month (black peak) and last month of analysis (white peak). MESF bead #4 was included as an example of the MESF kit used to control instrument performance during the 18 months of evaluation. The A4 conjugate was coupled later than the other conjugates and its intensities evaluation started at month 4.

The conjugate studies by conventional flow cytometry are shown in Fig 5. Fig 5A and 5B show the evaluation of each conjugate during 18 months. We observed a decrease in the fluorescence intensity (FI) of all conjugates studied. Among the anti-IgG-PE conjugates, A1 presented only 30% of decrease, while A2 achieved approximately 79% of decrease from the first to the last point of analysis. Regarding the stability of these conjugates, initial minus final fluorescence intensity (ΔFI) of A1 conjugate was the smallest value (ΔFI A1 = 490) when compared to other A conjugates coupled at the same time (ΔFI A2 = 988, ΔFI A3 = 718) and it was considered, therefore, the most stable one (Fig 5A). The behavior observed for the other set of anti-IgG-PE conjugates (Fig 5B) were similar to that seen on Fig 5A, however B were brighter (FI = 4564) then A conjugates (FI = 1656). Moreover, B conjugates were more stable than A2 and A3 and similar to A1 and A4. Among anti-HBSAg-PE, the D conjugate showed 48% of decrease, while the C conjugate decreased 60% during the analysis period (Fig 5B). Again, the behavior of both C and D conjugates were similar, but D was brighter (FI = 2569) than C (FI = 1770). The calculation of ΔFI for both C and D conjugates resulted in the observation that the last was more stable than the first one (Fig 5B). Fig 5C shows original flow cytometry histograms of all conjugates on the first point of evaluation (black peak) and on the last one (white peak). The decrease of fluorescence intensity on the comparative histograms was observed by the shift to the left detected for all conjugates, but with different intensities.

## Discussion

The use of microspheres (beads) as solid support for multiplex reactions is well described. There is a strong trend towards the employment of multiplexed diagnostic methods in various clinical demands, such as detection of HLA antigens for transplants, chronic leukemias, microbiological diagnostic, and allergies as well as detection of hepatitis C virus [25, 28, 29]. The combination of microspheres and flow cytometry as a tool for diagnosis has not been solely used in the identification of human diseases. Recently, Sousa *et al*. [30] proposed a serological method for canine leishmaniasis diagnosis using microspheres coupled with *Leishmania* antigens. Coupling antigens or antibodies to these microspheres is the basis of such methodology. This process is rather fragile and susceptible to variations due to intrinsic factors, such as the nature of the proteins to be coupled, as well as the conditions and reagents used in this process [31]. In fact, the analysis of microspheres using flow cytometry has been well established for many approaches [32–34], but its use in quality control of fluorescent conjugates is a novel purpose indeed. Since these fluorescent conjugates are the reporter antibodies in multiplex reactions, the quality control of such compounds is clearly essential. In quantitative methods, any changes in the reporter antibody can be normalized as the samples are plotted in the standard curves, but calibration curves still present random variability between batches [11]. In qualitative methods, there is no calibration curve to normalize samples and for this reason unobserved changes in the reporter conjugates may occur and lead to false results. Moreover, despite the type of assay, qualitative or quantitative, it is essential to establish efficient methods for quality control of reagents and processes involved in multiplex immunoassay reactions to assure their precision either in diagnostic or research fields [34, 35]. We thought that flow cytometry could be used in the quality control of immunoassays, whenever the principle of such assays is the detection of photons emitted after the excitation of a fluorochrome. We, therefore, have developed a protocol to couple these fluorescent conjugates to microspheres and tested the feasibility of such approach in the quality control of conjugates used in immunoassays.

The data presented in this manuscript showed that the intensities of different populations (codes) of microspheres did not interfere in the signal of the coupled antibody. Indeed, in another study, it was stated that bead classification fluorochromes have no spectral overlapping with the reporter fluorochrome PE [34], thus confirming our results. An optimal concentration of conjugate, however, must be established because it may affect the homogeneity of the coupling and consequently the quality control studies. In fact, the determination of the best reporter concentration is as an essential step for the development of diagnostic kits as described by several protocols [11, 31]. Our results showed that a clear narrow curve was observed at the highest concentration and concomitantly a lower coefficient of variation (CV) was detected when the profile of the histograms obtained with the lowest and the highest concentrations of anti-IgG-PE conjugates were compared. Thus, a small CV can be used as a parameter to determine the optimal conjugate concentration since it represents the most homogenous coupling process. The findings indicating that optimal coupling of antibodies to beads is a concentration-dependent process pointed out that the use of antibodies as reporter conjugates might be even more susceptible to variations, considering that beads are standardized materials. This observation reinforces the necessity of controlling these antibodies before using them on other procedures, as they are key reagents for the success of multiplex kits [35]. The possibility of evaluating these conjugates in a unique procedure designed for these purpose excludes the variations that may occur if the same conjugates are evaluated in a multiplex reaction, where they are used as the reporter antibody. In this context, Ellington *et al*. discussed the need of new quality control procedures in multiplex immunoassays, since processes applied in this kind of technique are susceptible to a great deal of variation [11].

Quantitative fluorescence cytometry (QFCM), in the context of quality control, could be a useful tool, since it intends to quantify molecules through flow cytometry and, therefore, is often taken as a promising method in the precise evaluation of fluorescence intensities, as well as variations between different instruments or laboratories [36]. Nowadays, QFCM is applied in instrument calibration and linearity verification [22], as well as in immunotherapy studies [18, 37], evaluation of membrane antigens expression in HIV [38] or leukemia patients [24, 39, 40], anti-HLA antibodies for kidney transplantation [25] and platelet-derived microparticles analysis [23]. There are different applications and manufacturers of microspheres for QFCM studies, as well as different types of evaluation that can be performed [22].

The establishment of instrument settings is considered the first step in order to achieve accurate QFCM evaluations [7]. Using this concept, in the present work we have adjusted the photomultiplier voltages at the first time point in order to obtain the expected histogram profiles of the five bead populations of the MESF PE kit, in accordance to the manufacturer recommendations. All the settings were maintained throughout the 18 months of the study, since any variation in the acquisition conditions on the flow cytometer could interfere in further QFCM analyses [7]. In fact, between the 4^th^ and the 7^th^ month of evaluation, we observed a decline in the fluorescence intensities of the beads. Since the overall analysis, excluding these months, demonstrated that the difference between the initial and final fluorescence values were small (157 for bead #4, 93 for bead #3, 23 for bead #2 and 1 for bead #1), we assumed that this decline was due to an alteration in the sensitivity of the instrument. Moreover, IgG-FITC labeling of microspheres coupled with anti-IgG-PE showed that no uncoupling or degradation of conjugates occurred. These findings support early statements about QFCM being an important tool to assure instrument linearity [26, 34] and also demonstrate how indispensable these quantitative measurements are for quality control in stability studies.

Concerning the stability evaluation for 18 months, we observed the same behavior in all conjugates studied, as well as for MESF PE kit beads. As discussed above, the fluorescence decline that was observed did not represent a real decrease in the fluorescent intensities of these conjugates. All conjugates showed a reduction in MESF values, during the evaluation period, differently from the MESF PE-kit beads. Taken together, these results indicate that the behavior of the MESF values of the conjugates represented a true decrease in the geometric means of these reagents during the time of the study. Since B2 and D conjugates showed a brighter profile, with geometric means exceeding the last possible point of the MESF curve, measuring these conjugates with the same settings under the same conditions of coupling was not possible. One possibility to solve this problem would be to test other dilutions. These could help by decreasing the intensities, but coupling conditions are altered concomitantly to the conjugate dilutions, as observed in this work. Thus, for overcoming this problem we determined that the performance and stability of the brightest conjugates should be evaluated by the traditional flow cytometry.

Regarding the brightness and stability of anti-IgG-PE conjugates, we observed a great variation in the fluorescent intensities (geometric means) between A and B manufacturers. B2 conjugate was brighter than all A conjugates, but A1 was the most stable. This difference between conjugates is one of the great concerns in immunoassays, since different capture and reporter antibodies can lead to different results [31]. These differences of brightness and stability between distinct conjugates were also observed in anti-HBSAg-PE studies where the D conjugate was brighter and more stable than the C conjugate. Moreover, a great variability between different batches was observed even within the same manufacturer. A1 was the most stable and the most brilliant among A conjugates. A2 and A3 conjugates had lower fluorescence intensities compared with A1, and also a greater decrease in their fluorescence intensities (79% and 77%, respectively) during the 18 months of analysis. Our data corroborate other previous reports, which stated that one should be especially careful when different batches of conjugates are used during the development of quality control methods of immunoassay reactions [26, 41].

Brightness and stability, indeed, are important characteristics to be taken into account when choosing fluorescent antibody to be used in immunoassays, since these characteristics affect directly the detection capacity of conjugates. Both characteristics depend on the fluorochrome used in the conjugation process. Several studies have been developed comparing brightness and photostability of different dyes/conjugates [42–45] or attempts to increase the sensitivity of detection [46]. Another process that affects these characteristics is conjugation between the fluorochrome and the protein. Gurvey *et al*. [46] had shown that the use of cyclodextrins can increase the brightness of the fluorochrome and that this is dependent on the type of the dye and on the fluorochrome/protein ratio (F/P ratio). F/P ratio can determine the number of fluorochromes conjugated to the antibody but it does not necessarily represent the fluorescence emission [7]. In this context, there is a latent need to develop more accurate methods to evaluate brightness and stability of antibodies for quality control purposes. Flow cytometry certainly provides more accurate data and new parameters for fluorescent intensities and stability analysis such as: histogram profiles, CVs evaluations, MESF analysis etc. Besides, the use of microspheres coupled with conjugates provides a direct measurement of PE emission and intensity, avoiding the variations inherent of multiplex reactions [11]. Altogether, this technique can be used not only to validate commercial antibodies applied in immunoassays but also to establish quality control protocols for the evaluation of *in house* conjugation processes.

## Conclusions

Our data showed that the proposed flow cytometric protocol using microspheres coupled with fluorescent conjugates can be applied as a standard quality control method in laboratories developing multiplex assays, by evaluating the performance and stability of conjugates used as reporter antibodies. We also showed that QFCM is an essential tool to guarantee the instrument linearity and sensibility status, in order to validate the results of stability tests.

## References

[1] Weiner LM, Surana R, Wang S. Monoclonal antibodies: versatile platforms for cancer immunotherapy. Nat Rev Immunol 2010; 10: 317–327. 10.1038/nri2744 20414205 PMC3508064

[2] Shapiro HM. Practical Flow Cytometry. 4th ed New York: John Wiley & Sons Inc.; 2003.

[3] Dos Santos RV, De Lima PPG, Nitsche A, Harth FM, De Melo FY, Akamatsu HT, et al Aplicações terapêuticas dos anticorpos monoclonais. Rev Bras Alerg Imunopatol 2006; 29: 77–85.

[4] Shukla AA, Hubbard B, Tressel T, Guhan S, Low D. Downstream processing of monoclonal antibodies–Application of platform approaches. J Chromatogr B 2007; 848: 28–39. 10.1016/j.jchromb.2006.09.026 17046339

[5] Li F, Vijayasankaran N, Shen AY, Kiss R, Amanullah A. Cell culture processes for monoclonal antibody production. Monoclonal Antibodies 2010; 2: 466–477. 10.4161/mabs.2.5.12720 20622510 PMC2958569

[6] Schmidt C. The purification of large amounts of monoclonal antibodies. J Biotechnol 1989; 11: 235–252.

[7] Vogt RF Jr, Whitfield WE, Henderson LO, Hannon WH. Fluorescence Intensity Calibration for Immunophenotyping by Flow Cytometry. Methods 2000; 21: 289–296. 10.1006/meth.2000.1009 10873483

[8] Lysák D, Kalina T, Martínek J, Pikalová Z, Vokurková D, Jaresová M, et al Interlaboratory variability of CD34+ stem cell enumeration. A pilot study to national external quality assessment within the Czech Republic. Int Jnl Lab Hem 2010; 32: 229–236. 10.1111/j.1751-553X.2010.01244.x 20561093

[9] Reilly JT. Use and evaluation of leukocyte monoclonal antibodies in the diagnostic laboratory: a review. Clin Lab Haem 1996; 18: 1–5. 10.1111/j.1365-2257.1996.tb00728.x 9118596

[10] Carey J, Oldaker TA. More than Just Quality Control. Clin Lab Med 2007; 27: 687–707. 10.1016/j.cll.2007.05.010 17658413

[11] Ellington AA, Kullo IJ, Bailey KR, Klee GG. Antibody-Based Protein Multiplex Platforms: Technical and Operational Challenges. Clin Chem 2010; 56: 186–193. 10.1373/clinchem.2009.127514 19959625 PMC2901849

[12] Wiczling P, Ait-Oudhia S, Krzyanski W. Flow cytometric analysis of reticulocyte maturation after erythropoietin administration in rats. Cytometry 2009; 75: 584–92. 10.1002/cyto.a.20736 19437531

[13] Barth T, Oliveira PR, D`Avila FB, Dalmora SL. Validation of the normocythemic mice bioassay for the potency evaluation of recombinant human erythropoietin in pharmaceutical formulations. J AOAC International 2008; 91: 285–91. 18476339

[14] Jung KM, Bae I-H, Kim B-H, KIM W-K, Chung J-H, Park Y-H, et al Comparison of flow cytometry and immunohistochemistry in non-radioisotopic murine lymph node assay using bromodeoxyuridine. Toxicol Lett 2010; 192: 229–37. 10.1016/j.toxlet.2009.10.024 19879932

[15] Bertho AL, Ferraz R. Citometria de Fluxo: princípios metodológicos de funcionamento In: Sales MM, Moraes-Vasconcelos D, editors. Citometria de Fluxo: aplicações no laboratório clínico e de pesquisa. São Paulo: Editora Atheneu; 2013 pp. 3–19.

[16] Comas-Riu J, Rius N. Flow cytometry applications in the food industry. J Ind Microbiol Biot 2009; 36: 999–1011. 10.1007/s10295-009-0608-x 19557445

[17] de Oliveira ERA, Lima BMMP, dos Santos BAF, de Moura WC. Nogueira ACMA. Potency determination of recombinant IFN-alpha based on phosphorylated STAT1 using flow cytometry. J Immunol Methods 2012; 375: 271–275. 10.1016/j.jim.2011.11.005 22115721

[18] Randlev B, Huang L-C, Watatsu M, Marcus M, Lin A, Shih S-J. Validation of a quantitative flow cytometer assay for monitoring HER-2/neu expression level in cell-based cancer immunotherapy products. Biologicals 2010; 38: 249–259. 10.1016/j.biologicals.2009.12.001 20080049

[19] Rigato PO, Brito CA. Quantificação de antígenos de superfície celular e ensaios multiplex In: Sales MM, Moraes-Vasconcelos D, editors. Citometria de Fluxo: aplicações no laboratório clínico e de pesquisa. São Paulo: Editora Atheneu; 2013 pp. 35–56.

[20] Henderson LO, Marti GE, Gaigalas A, Hannon WH, Vogt RF Jr. Terminology and Nomenclature for Standardization in Quantitative Fluorescence Cytometry. Cytometry 1998; 33: 97–105. 9773869 10.1002/(sici)1097-0320(19981001)33:2<97::aid-cyto3>3.0.co;2-h

[21] Schwartz A, Wang L, Early E, Gaigalas A, Zhang Y-Z., Marti GE, et al Quantitating Fluorescence Intensity from Fluorophore: The Definition of MESF Assignment. J Res Natl Inst Stand Technol 2002; 107: 83–91. 10.6028/jres.107.009 27446720 PMC4865278

[22] Mittag A, Tárnok A. Basics of standardization and calibration in cytometry- a review. J Biophoton 2009; 2: 470–481. 10.1002/jbio.200910033 19504519

[23] Mobarrez F, Antovic J, Egberg N, Hansson M, Jörneskog G, Hultenby K, et al A multicolor flow cytometric assay for measurement of platelet-derived microparticles. Thromb Res 2010; 125: 110–116. 19939440 10.1016/j.thromres.2009.10.006

[24] Kay S, Herishanu Y, Pick M, Rogowski O, Baron S, Naparstek E, et al Quantitative Flow Cytometry of ZAP-70 Levels in Chronic Lymphocytic Leukemia Using Molecules of Equivalent Soluble Fluorochrome. Cytometry Part B 2006; 70B: 218–226. 10.1002/cyto.b.20078 16456869

[25] Ishida H, Hirai T, Kohei N, Yamaguchi Y, Tanabe K. Significance of qualitative and quantitative evaluations of anti-HLA antibodies in kidney transplantation. Transplant Int 2010; 24: 150–157. 10.1111/j.1432-2277.2010.01166.x 21208294

[26] Gratama JW, D`Hautcourt J-L, Mandy F, Rothe G, Barnett D, Janossy G, et al Flow Cytometric Quantitation of Immunofluorescence Intensity: Problems and Perspectives. Cytometry 1998; 33: 166–178. 9773877 10.1002/(sici)1097-0320(19981001)33:2<166::aid-cyto11>3.0.co;2-s

[27] Wang L, Gaigalas AK, Marti G, Abassi F, Hoffman RA. Toward Quantitative Fluorescence Measurements with Multicolor Flow Cytometry. Cytometry Part A 2008; 73A: 279–288. 10.1002/cyto.a.20507 18163471

[28] Fonseca BPF, Marques CFS, Nascimento LD, Mello MB, Silva LBR, Rubim NM, et al Development of a Multiplex Bead-Based Assay for Detection of Hepatitis C Virus. Clin Vaccine Immunol 2011; 18: 802–806. 10.1128/CVI.00265-10 21346054 PMC3122539

[29] Hsu H-Y, Joos TO, Koga H. Multiplex microsphere-based flow cytometric platforms for protein analysis and their application in clinical proteomics–from assays to results. Electrophoresis 2009; 30: 4008–4019. 10.1002/elps.200900211 19960465

[30] Sousa S, Cardoso L, Reed SG, Reis AB, Martins-Filho OA, Silvestre R, et al Development of a Fluorescent Based Immunosensor for the Serodiagnosis of Canine Leishmaniasis Combining Immunomagnetic Separation and Flow Cytometry. PLoS Negl Trop Dis 2013; 7: e2371, 2013. 10.1371/journal.pntd.0002371 23991232 PMC3749986

[31] Elshal MF, McCoy JP. Multiplex bead array assays: performance evaluation and comparison of sensitivity to ELISA. Methods 2006; 38: 317–323. 10.1016/j.ymeth.2005.11.010 16481199 PMC1534009

[32] Vignali DAA. Multiplexed particle-based flow cytometric assays. J Immunol Methods 2000; 243: 243–255. 10986418 10.1016/s0022-1759(00)00238-6

[33] Jani I, Janossy G, Brown DW, Mandy F. Multiplexed immunoassays by flow cytometry for diagnosis and surveillance of infectious diseases in resources-poor settings. The Lancet 2002; 2: 243–250. 11937424 10.1016/s1473-3099(02)00242-6

[34] Kellar KL, Iannone MA. Multiplexed microsphere-based flow cytometric assays. Experimental Hematology 2002; 30: 1227–1237. 12423675 10.1016/s0301-472x(02)00922-0

[35] Tighe P, Negm O, Todd I, Fairclough L. Utility, reliability and reproducibility of immunoassay multiplex kits. Methods 2013; 61: 23–29. 10.1016/j.ymeth.2013.01.003 23333412

[36] Pannu KK, Joe ET, Iyer SB. Performance Evaluation of QuantiBRITE Phycoerythrin Beads. Cytometry 2001; 45: 250–258. 11746094 10.1002/1097-0320(20011201)45:4<250::aid-cyto10021>3.0.co;2-t

[37] Chan HEH, Jilani I, Chang R, Albitar M. Quantification of Surface Antigens and Quantitative Flow Cytometry. Methods Mol Biol 2007; 378: 65–69. 10.1007/978-1-59745-323-3_5 18605078

[38] Liu Z, Cumberland WG, Hultin LE, Prince HE, Detels R, Giorgi JV. Elevated CD38 antigen expression on CD8+ T cells is a stronger marker for the risk of chronic HIV disease progression to AIDS and death in the Multicenter AIDS Cohort Study than CD4+ cell count, soluble immune activation markers, or combinations of HLA-DR and CD38 expression. J Acquir Immune Defic Syndr Hum Retrovirol 1997; 16:83–92. 9358102 10.1097/00042560-199710010-00003

[39] Wang L, Abassi F, Gaigalas A, Vogt RF, Marti GE. Comparision of Fluorescein and Phycoerythrin Conjugates for Quantifying CD20 Expression on Normal and Leukemic B-Cells. Cytometry Part B 2006; 70B: 410–415. 10.1002/cyto.b.20140 16967494

[40] Rossman ED, Lenkei R, Lundin J, Mellstedt H, Österborg A. Performance of Calibration Standards for Antigen Quantitation with Flow Cytometry in Chronic Lymphocytic Leukemia. Cytometry Part B 2007; 72B: 450–457. 10.1002/cyto.b.20359 17565749

[41] Voskuil JLA. Commercial antibodies and their validation. F1000 Research. 2014; 10 02. 10.12688/f1000research.4966.1 PMC4197739 25324967

[42] Patsenker L, Tartarets A, Kolosova O, Obukhova O, Povrozin Y, Fedyunyayeva I, et al Fluorescent Probes and Labels for Biomedical Applications. Ann. N.Y. Acad. Sci. 2008; 1130: 179–187. 10.1196/annals.1430.035 18596347

[43] Mahmoudian J, Hadavi R, Jeddi-Tehrani M, Mahmoudi AR, Bayat AA, Shaban E, et al Comparision of the Photobleaching and Photostability Traits of Alexa Fluor 568- and Fluorescein Isothiocyanate- conjugated Antibody. Cell Journal 2011; 13: 169–172. 23508937 PMC3584473

[44] Hayashi-Takanaka Y, Stasevich T, Kurumizaka H, Nozaki N, Kimura H. Evaluation of Chemical Fluorescent Dyes as a Protein Conjugation Partner of Live Cell Imaging. PLoS ONE 2014; 9: e106271 10.1371/journal.pone.0106271 25184362 PMC4153647

[45] Munier M, Jubeau S, Wijaya A, Morançais M, Dumay J, Marchal L, et al Physicochemical factors affecting the stability of two pigments: R-phycoerythrin of *Grateloupia turuturu* and B-phycoerythrin of *Porphyridium cruentum*. Food Chem 2014; 150: 400–407. 10.1016/j.foodchem.2013.10.113 24360468

[46] Gurvey O, Abrams B, Lomas C, Nasraty Q, Park E, Dubrovsky T. Control of the Fluorescence of Dye-Antibody Conjugates by (2-Hydroxypropyl)-ß-cyclodextrin in Fluorescence Microscopy and Flow Cytometry. Anal Chem 2011; 83: 7109–7114. 10.1021/ac2014146 21846137

